# Mitral Valve Annuloplasty Ring Dehiscence Diagnosed Intraoperative With Real-Time 3D Transesophageal Echocardiogram

**DOI:** 10.1177/2324709614538822

**Published:** 2014-06-24

**Authors:** Karina Castellon-Larios, Alix Zuleta-Alarcon, Antolin Flores, Michelle Humeidan, Andrew N. Springer, Michael Essandoh

**Affiliations:** 1 The Ohio State University Wexner Medical Center, Columbus, OH, USA

**Keywords:** mitral valve calcification, annuloplasty ring dehiscence, 3D echocardiography

## Abstract

Mitral annular calcification (MAC) is often a result of the accumulation of lipids around the annulus, which can lead to degeneration and calcification of the valve. Multiple risk factors have been associated with the progression of MAC and life-threatening complications such as the early mitral valve annuloplasty dehiscence. Our case describes the different risk factors for annuloplasty dehiscence in a patient with severe MAC, as well as the importance of its early recognition intraoperatively with 3D transesophageal echocardiography.

## Introduction

Mitral regurgitation (MR) and mitral annular calcification (MAC) are common conditions in middle-aged and elderly population with a prevalence of 2% and 8%, respectively. Mitral valve regurgitation is commonly associated with left ventricular remodeling, secondary to coronary artery disease, and hypertension, among others. MAC risk factors include both factors mentioned above, as well as Caucasian race, female gender, hyperlipidemia, diabetes, current or past history of smoking, atherosclerosis, chronic renal insufficiency, and high levels of interleukin-6, which may lead to all-cause mortality.^1-3^

Mitral valve repair is the treatment of choice for MR and leads to better preservation of left ventricular function and survival.^4^ Real-time 3D transesophageal echocardiogram provides a detailed image of mitral valve pathology, mechanism of MR, and severity.^5^ This better understanding of the MR pathophysiology and the alteration of annular geometry has contributed to better repair techniques and the placement of etiology-specific rings, which are specially challenging when MR is accompanied by MAC.^4^

## Case Presentation

A 69-year-old Caucasian female with a past medical history significant for polycystic kidney disease requiring a cadaveric kidney transplant, hypertension, hyperlipidemia, coronary artery disease and MR, was transferred to our institution for triple vessel coronary artery bypass graft (CABG) and mitral valve repair.

She had presented to an outside hospital with angina at rest and worsening dyspnea on exertion. She underwent cardiac catheterization, which showed severe multivessel coronary artery disease, including a nearly completely occluded right coronary artery with some collateral filling from the left, high-grade proximal left anterior descending (LAD) lesion, as well as circumflex arterial lesions in the first and second obtuse marginals.

Transthoracic echocardiogram performed at our institution revealed moderate to severe left ventricular systolic dysfunction with an estimated ejection fraction of 25% to 30%, diastolic filling pattern consistent with Grade II diastolic dysfunction (pseudonormalization), moderate concentric left ventricular hypertrophy, regional wall motion abnormalities, moderate ischemic MR, and moderate to severe MAC.

The patient was taken to the operating room and general anesthesia was induced uneventfully under multichannel invasive monitoring. Intraoperative transesophageal echocardiogram (TEE) was performed with a 3D matrix-array probe (X7-2t transducer; Philips Healthcare, Andover, MA) that showed similar results as the preoperative transthoracic echocardiogram except for moderate to severe central MR.

**Figure 1.**
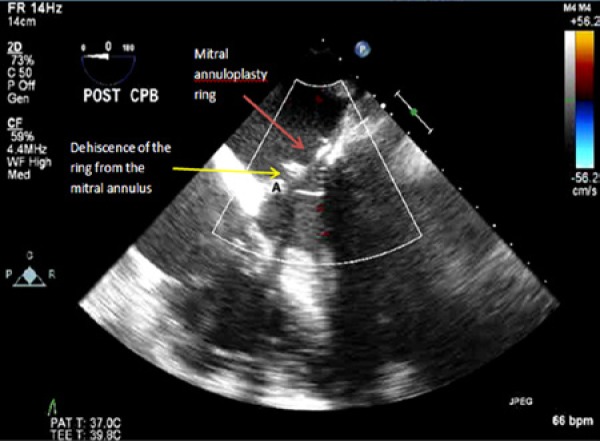
Two-dimensional transesophageal echocardiography midesophageal 4-chamber view showing dehiscence of the annuloplasty ring. Abbreviation: A, mitral annulus.

**Figure 2.**
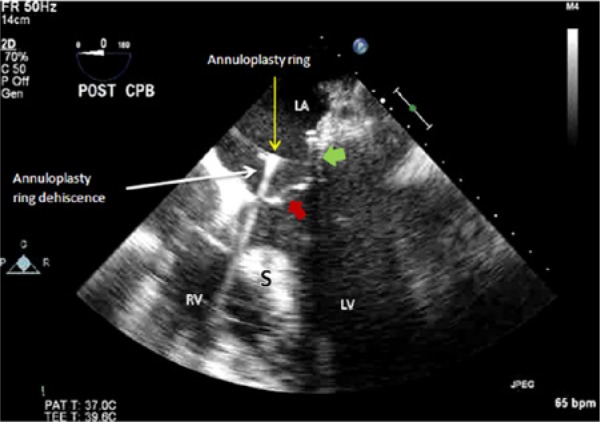
Two-dimensional transesophageal echocardiography midesophageal 5-chamber view showing dehiscence of the annuloplasty ring, with the ring causing echo dropout. The red arrow shows the anterior mitral valve leaflet, and the green arrow shows the posterior mitral valve leaflet. Abbreviations: S, interventricular septum; RV, right ventricle; LV, left ventricle.

**Figure 3.**
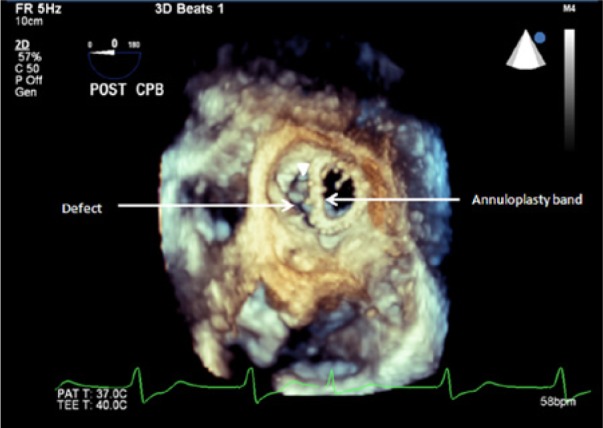
En face view 3D transesophageal echocardiography illustrating the anterior defect of the annuloplasty ring at the aorto-mitral curtain, through the defect the precise location of the anterior mitral valve leaflet is readily apparent (arrow head).

The patient underwent an uneventful triple vessel CABG (left internal mammary artery to her LAD, saphenous vein graft to a dominant obtuse marginal, and saphenous vein graft to her acute marginal), as well as a mitral valve repair with a size 26 mm St Jude annuloplasty ring. Post bypass 2D TEE assessment showed an echodense structure in the mitral annulus close to the aorto-mitral curtain without any MR on color flow Doppler (Figures 1 and 2). The absence of MR was likely due to occlusion of the orifice between the annulus and the ring during systole by the anterior mitral valve leaflet. Three-dimensional TEE was then performed, revealing partial ring dehiscence and deformation of the annuloplasty ring along the anterior aspect of the mitral annulus. A clear diagnosis of mitral valve annuloplasty dehiscence was made (Figure 3).

## Discussion

Chronic renal insufficiency has been shown to promote calcium accumulation in cardiovascular structures, including the mitral annulus in up to 31% of patients.^6^ Movva et al. systemically evaluated the mitral valve and annular calcification in patients with chronic kidney disease and on hemodialysis. They observed MAC to be prevalent in this patient population and found it to be associated with MR than MS.^7^

### The Role of Echocardiography in Mitral Pathology

Real-time 3D TEE is a valuable tool in the diagnosis of heart disease, especially for mitral valve pathology, its optimal localization, extension, and mechanism.^8^ Kronzon et al. demonstrated that 3D TEE provides detailed information regarding the size, shape, and area of the dehisced segment, when compared with 2D TEE in the diagnosis of mitral valve annuloplasty and prosthetic valve dehiscence.^9^ In other clinical scenarios, 3D TEE has been shown to be accurate and concordant with surgical and catheterization findings in mitral valve stenosis orifice area calculation, functional anatomy of MR, and evaluation of prolapsing mitral valve scallops. Furthermore, in the diagnosis of complex pathologies, 3D TEE also provides optimal spatial resolution and capability to precisely describe the characteristics of native and prosthetics valves.^8,10,11^

In our case, 3D TEE allowed for a precise intraoperative diagnosis of a segmental separation between the native mitral valve annulus and the prosthetic ring at the aorto-mitral curtain. Three-dimensional TEE simplifies communication and visualization of echocardiographic findings between the anesthesiology and surgical teams. In emergent as well as nonemergent procedures, it provides a prompt and accurate diagnosis of mitral valve pathology for immediate decision making and treatment.^12^ Real-time 3D TEE constitutes a powerful tool to plan the appropriate interventional approach and identify concomitant cardiac pathologies. ^9,13^

## Conclusion

Although 2D TEE is currently the standard of care for intraoperative assessment after valve surgery, especially mitral valve pathology, it is well documented throughout the literature that there are several limitations to this approach.^14^ Real-time 3D TEE is a new approach to intraoperative assessment after valve surgery and is still not considered standard of care for these or any other type of procedures. Nonetheless, it is a very accurate method for detection of intraoperative complications, including annuloplasty dehiscence, when the 2D TEE results are inconclusive.
